# A common language to assess allergic rhinitis control: results from a survey conducted during EAACI 2013 Congress

**DOI:** 10.1186/s13601-015-0080-9

**Published:** 2015-10-27

**Authors:** Peter W. Hellings, Antonella Muraro, Wytske Fokkens, Joaquim Mullol, Claus Bachert, G. Walter Canonica, David Price, Nikos Papadopoulos, Glenis Scadding, Gerd Rasp, Pascal Demoly, Ruth Murray, Jean Bousquet

**Affiliations:** Department of Otorhinolaryngology, University Hospitals Leuven, Leuven, Belgium; Department of Otorhinolaryngology, Academic Medical Center (AMC), Amsterdam, The Netherlands; Department of Women and Child Health, Food Allergy Referral Centre, Padua University Hospital, Veneto Region, Padua, Italy; Hospital Clinic, IDIBAPS, CIBERES, Barcelona, Catalonia Spain; Upper Airways Research Laboratory (URL), University Hospital Ghent, Ghent, Belgium; Allergy and Respiratory Diseases, Department of Internal Medicine, IRCCS S Martino, IST, University of Genoa, Genoa, Italy; Centre of Academic Primary Care, University of Aberdeen, Aberdeen, UK; Allergy Department, 2nd Pediatric Clinic, University of Athens, Athens, Greece; RNTNE Hospital, London, UK; Department of Otorhinolaryngology, Paracelsus Medical University, Salzburg, Austria; Division of Allergy, Department of Pulmonology, Hôpital Arnaud de Villeneuve, University Hospital of Montpellier, Montpellier, France; MedScript Ltd, Dundalk, Co. Louth, Ireland; University Hospital, Montpellier, France; MACVIA-LR, Contre les Maladies Chronique pour un Vieillissement Actif en Languedoc Roussilon, European Innovation Partnership on Active and Healthy Aging Reference Site, Montpellier, France; INSERM, VIMA: Ageing and Chronic Diseases. Epidemiological and Public Health Approaches, U1168 Paris, France; UVSQ UMR-S1168, Universite Versailles St-Quentin-en-Yvelines, Versailles, France

**Keywords:** Allergic rhinitis, Control, Digital, Survey, Visual analogue scale, VAS

## Abstract

**Background:**

The concept of control is gaining importance in the field of allergic rhinitis (AR), with a visual analogue scale (VAS) score being a validated, easy and attractive tool to evaluate AR symptom control. The doctors’ perception of a VAS score as a good tool for evaluating AR symptom control is unknown, as is the level of AR control perceived by physicians who treat patients.

**Methods:**

307 voluntarily selected physicians attending the annual (2013) European Academy of Allergy and Clinical Immunology (EAACI) meeting completed a digital survey. Delegates were asked to (1) estimate how many AR patients/week they saw during the season, (2) estimate the proportion of patients they considered to have well-, partly- and un-controlled AR, (3) communicate how they gauged this control and (4) assess how useful they would find a VAS as a method of gauging control. 257 questionnaires were filled out completely and analysed.

**Results:**

EAACI delegates reported seeing 46.8 [standard deviation (SD) 68.5] AR patients/week during the season. They estimated that 38.7 % (SD 24.0), 34.2 % (SD 20.2) and 20.0 % (SD 16.34) of their AR patients had well-controlled (no AR symptoms), partly-controlled (some AR symptoms), or un-controlled-(moderate/severe AR symptoms) disease despite taking medication [remainder unknown (7.1 %)]. However, AR control was assessed in many ways, including symptom severity (74 %), frequency of day- and night-time symptoms (67 %), activity impairment (57 %), respiratory function monitoring (nasal and/or lung function; 40 %) and incidence of AR exacerbations (50 %). 91 % of delegates felt a simple VAS would be a useful tool to gauge AR symptom control.

**Conclusions:**

A substantial portion of patients with AR are perceived as having uncontrolled or partly controlled disease even when treated. A simple VAS score is considered a useful tool to monitor AR control.

**Electronic supplementary material:**

The online version of this article (doi:10.1186/s13601-015-0080-9) contains supplementary material, which is available to authorized users.

## Background

Control of disease is considered one of the key outcomes in several medical domains. Although the concept of control is well-defined in asthma, chronic obstructive pulmonary disease and other conditions such as glycaemic control in diabetes, [1] it has only recently gained significant attention in the field of allergic rhinitis (AR) [2–5]. Indeed, the patients’ evaluation of disease control by any type of treatment, leading to a significant reduction of symptom severity, has become one of the novel goals of treatment in different chronic diseases. In AR, there is growing consensus that a visual analogue scale (VAS) score represents a simple, good and valid tool to monitor AR disease control [2, 6]. In 2010, Bousquet and colleagues [7] proposed a simple VAS to evaluate AR control. More sophisticated means of monitoring AR control have been used without showing superiority of one over the other [8]. Therefore, a VAS has been incorporated into the treatment algorithms for AR [2] to guide treatment decisions as part of an integrated care pathway [9]. It has yet to be validated in children. Nowadays, a digital version of the AR control VAS will be rolled out to patients, pharmacists and physicians to encourage better communication (with patients) and referral when appropriate. Physicians of all specialities involved in AR management can use the same VAS, from general practitioners (GPs) and allergists, to ear nose and throat (ENT) specialists, paediatricians, pulmonologists and dermatologists.

A VAS for AR has been shown to assess disease severity according to the Allergic Rhinitis and its Impact on Asthma (ARIA)-guidelines, with a VAS cut off score of 50 mm distinguishing between mild and moderate/severe AR in adults [10]. The VAS score incorporates quality of life (QoL) and reflective total nasal symptom score (rTNSS) [11] and correlates with improvements in AR symptoms and QoL. It can be used to assess AR severity in both intermittent and persistent disease in untreated or treated patients [10]. A change in VAS score of more than 23 mm represents clinically relevant changes in QoL and AR symptoms, possibly reflecting a response to treatment [6].

The major gaps in the current appreciation of VAS as a tool for the evaluation of symptom control are the following: (1) the level of control reached in patients by actual treatment options as perceived by the medical doctors, (2) how disease control is evaluated, and (3) physician perception on the usefulness of a VAS score for the evaluation of symptom control. Physicians often under-estimate disease severity and impact on patients’ lives, while at the same time over-estimate the effectiveness of treatment [12, 13]. Physician-patient communication is greatly hampered by a lack of a common language to describe AR control and a lack of a universally-accepted definition of what AR control actually means. The aim of this exploratory study was to assess how physicians measure AR symptom control, how they perceive the control status of their patients and how they regard the usefulness of a VAS to gauge disease control.

## Methods

A quantitative, digital survey, designed to collect views of physicians who treat AR routinely in clinical practice, was carried out during the 32nd EAACI Congress (Milan, Italy) from 22nd to 26th June 2013. The survey content was informed by experts in the field of AR (JB, CB and DP) and conducted at the Meda booth by physicians attending the exhibition (see Additional file 1). There was no incentive to take part in the survey.

Those who consented to take part had their EAACI barcode scanned, were allocated a digital ID and were provided with the survey questions on an iPad. Responses to all questions were anonymised and stored on an independent server.

Delegates were asked to:

1. Estimate how many AR patients they saw per week during the season,
2. estimate the proportion of their patients they considered to have well-, partly- and un-controlled disease,
3. communicate how they gauged this control (>1 answer permitted)
4. assess how useful they would find a VAS as a method of gauging control.

A representation of a VAS with marker slider was shown to delegates when considering their response to question 4. Survey questions and representation of the VAS with marker slider are provided in Additional file 1.

Descriptive statistics (mean and standard deviation) were used to summarise survey responses.

## Results

307 EAACI 2013 delegates from 60 different countries and from different specialties (e.g. GPs, allergists, ENT specialists and paediatricians) completed the survey. Valid question responses were obtained for 257 of these. Surveys from 50 delegates were not included as they were incomplete.

**Fig. 1:**
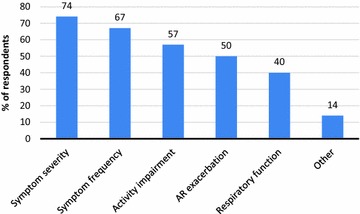
Methods used by EAACI 2013 meeting delegates to assess AR symptom control. Respiratory function monitoring refers to nasal and/or lung function

On average, respondents reported seeing 46.8 [standard deviation (SD) 68.5] AR patients/week during the season. They estimated that AR was well-controlled, partly-controlled and un-controlled in 38.7 % (SD 24.0), 34.2 % (SD 20.2) and 20.0 % (SD 16.34) of patients, respectively, and unknown for the remainder (7.1 %). Delegates reported assessing disease control in many different ways, including symptom severity (74 %), frequency of day- and night-time symptoms (67 %), activity impairment (57 %), respiratory function monitoring (nasal and/or lung function; 40 %) and incidence of AR exacerbations (50 %) (Fig. 1). 91 % of delegates felt that a VAS was a useful tool to assess disease control.

## Discussion

According to 257 EACCI 2013 delegates, the VAS score is a useful tool to monitor disease control in AR. More than 50 % of AR patients were considered by physicians to have partly-controlled or uncontrolled disease, with many different features of AR, unrelated to nasal symptoms, determining physicians’ perception of disease of control. This observation that >50 % of their patients have sub-optimal AR control is in agreement with other surveys [13–15]. The physicians’ perception of reaching a good level of control in 38.7 % of patients also corresponds well with previous reports: A European survey found that, according to physician assessment, good control of nasal and ocular symptoms was achieved in 45.4 and 51.3 % of patients, respectively [13].

It was also apparent that AR control was assessed in multiple ways with no consensus on an optimal method. Of note was the large standard deviation seen around control perception, which shows a wide-ranging response to the question. Interestingly, the determinants of control as perceived by the physicians varied from frequency of day- and night-time symptoms to respiratory function monitoring (nasal and/or lung function). One of the most striking findings was the extra-nasal symptoms, like frequency of day- and night-time symptoms and impaired activity being reported as key determinants of AR control.

91 % of the EAACI delegates who completed this survey agreed upon the validity of a VAS as a useful tool for assessing AR control. In our opinion, it is an intuitive tool for use in clinical studies, and by physicians and patients every day. The VAS is well suited to the task of assessing AR control. It is simple and quick to complete, incorporating assessment of both AR symptoms and quality-of-life [11]. It correlates well with recognized randomized controlled trial endpoints [16], can discriminate according to severity [10] and assess efficacy of treatments [16, 17]. The VAS has also been used to assess effectiveness of treatments in real-life [18] as well as inadequacies of others (including multiple treatments) [19].

Limitations of this survey relate to the fact that the most respondents were specialists (although with experience in treating AR), with relatively few GPs included. Delegates were not provided with an alternative control tool choice and also completed the survey at the Meda booth, which may have introduced bias. However, no financial or any other incentive was given to complete the survey. Also delegate speciality was not consistently recorded which may have yielded interesting insights into how AR control is assessed across specialities. Finally, information on what proportion of AR patients had concomitant asthma was not captured. This would have provided important information on how disease control was assessed and whether the perception of control was better or worse for those patients with co-morbid disease.

The VAS will form the basis of a new contre les MAladies Chronique pour un VIeillissement Actif (MACVIA)-ARIA AR app directed at patients called ‘*Allergy Diary*’ which is now available for free download in many European countries. Users can assess their disease control daily by simply clicking on the VAS in response to the question ‘overall how much are your allergic symptoms bothering you today?’, from ‘not at all bothersome’ to ‘extremely bothersome’. VAS scores are logged and plotted over time with control assessed as well-, partly- and un-controlled, according to specific VAS score cut-offs. The VAS will also be incorporated into a companion app for healthcare providers as well as into the new AR guideline, and used to guide treatment decisions. Moving to a digital VAS is attractive since in real life, on paper, VAS scores are often wrongly completed by the patient, even after explanation; either by failing to cross the line, putting a cross above or below it or writing a figure. An electronic version would prevent such errors. However, it may not allow for complexity of response such as persistence of a problematical co- morbidity despite good control of AR. The overall aim of ‘*Allergy Diary*’, the Allergy Diary companion app and the updated AR guideline (and the VAS contained within them) is to facilitate a top down communication, from guidelines to healthcare providers to patients, allowing doctors to more easily comply with the guidelines, to better tailor AR medications to patients’ needs and enable patients to better communicate their needs.

In short, a common language of AR disease control is needed. A simple VAS to measure and assess disease control could meet this need and is welcomed by physicians. It should enable us to move from the illusion to the confirmation of communication.

## Additional file

10.1186/s13601-015-0080-9 Survey questions. The questions asked in a quantitative, digital survey carried out during the 32nd EAACI Congress (Milan, Italy) from 22nd to 26th June 2013.The survey was designed to collect views of physicians who treat AR routinely in clinical practice. It includes a representation of a VAS with maker slider that was shown to delegates when considering their response to question 9 of the survey.

## Abbreviations

AR: allergic rhinitis
ARIA: allergic rhinitis and its impact on asthma
EAACI: European Academy of Allergy and Clinical Immunology
ENT: ear nose and throat
GP: general practitioner
MACVIA: Contre les MAladie Chronique pour un VIeillissement Actif
QoL: quality of life
rTNSS: reflective total nasal symptom score
SD: standard deviation
VAS: visual analogue scale
