# Supplementary material for: A common language to assess allergic rhinitis control: results from a survey conducted during EAACI 2013 Congress
Source: Clin Transl Allergy. 2015 Oct 27;5:36. doi: 10.1186/s13601-015-0080-9 (PMC4621924; doi:10.1186/s13601-015-0080-9)

**Survey questions**

1. Name
2. Surname
3. Country
4. Email
5. Practice centre
6. How many AR patients do you see in your practice in a given week during the season?
7. What proportion of your AR patients are:
   1. Well-controlled (i.e. have no AR symptoms when taking their medication)?
   2. Partly controlled (i.e. have some AR symptoms when taking their medication)?
   3. Uncontrolled (i.e. have moderate/severe AR symptoms despite taking an intranasal corticosteroid)
8. How do you assess AR symptom control in your clinical practice? (tick all that apply)
   1. Symptom severity
   2. Frequency of daily/nocturnal symptoms
   3. Level of activity impairment
   4. Respiratory function monitoring
   5. Allergic rhinitis exacerbations (i.e. unscheduled medical consultation and rescue medication use)
   6. Other
9. Would you find a simple visual analogue scale useful to assess AR symptom control?
   1. Yes
   2. No

**A representation of a VAS with maker slider that was shown to delegates when considering their response to question 9 of the survey**


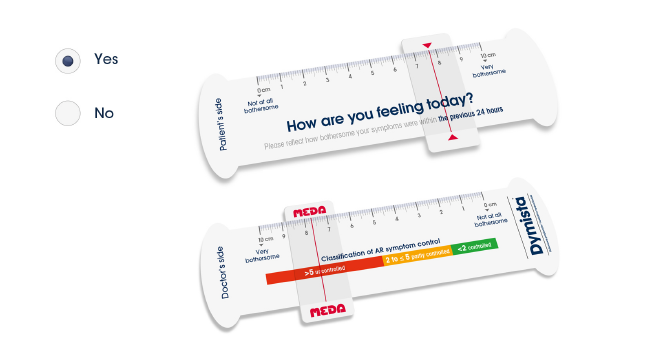

Supplement: Supplementary file 1 — 10.1186/s13601-015-0080-9 Survey questions. The questions asked in a quantitative, digital survey carried out during the 32nd EAACI Congress (Milan, Italy) from 22nd to 26th June 2013.The survey was designed to collect views of physicians who treat AR routinely in clinical practice. It includes a representation of a VAS with maker slider that was shown to delegates when considering their response to question 9 of the survey. [file 13601_2015_80_MOESM1_ESM.docx]
